# A Case of Concurrent Infection With Syphilis and Monkeypox in an Immunosuppressed Individual

**DOI:** 10.7759/cureus.41690

**Published:** 2023-07-11

**Authors:** Ramya Pakala, Faisal Syed, Anna C Pinelo, Gagan Singh, Jhansi Gajjala

**Affiliations:** 1 Internal Medicine, Howard University Hospital, Washington, USA; 2 Infectious Disease, Howard University Hospital, Washington, USA

**Keywords:** hiv aids, tecovirimat, human immunedeficiecy virus (hiv) infection, syphilis, monkeypox

## Abstract

Monkeypox and syphilis are two distinct infectious diseases that can cause severe health complications in infected patients and can share clinical manifestations, making their simultaneous occurrence challenging to diagnose and manage. Here, we present a case report of a patient with the coinfection of monkeypox and syphilis, highlighting the clinical presentation and treatment considerations. This case underlines the importance of considering coinfections in patients presenting with atypical clinical manifestations and risk factors. This case highlights the importance of early diagnosis and prompt intervention in managing patients with infectious diseases, particularly when dealing with multiple infections. Increased awareness among healthcare professionals regarding the potential for concurrent infections can enhance diagnostic accuracy and improve patient care in similar challenging cases.

## Introduction

A monkeypox (MPX) outbreak was reported by the WHO in non-endemic areas in May 2022 [1]. MPX presents initially with skin changes that can be confused with other viral or bacterial diseases. This can lead to additional challenges in diagnosing and managing such cases. We present a unique case of concurrent infection with overlapping symptom presentations of syphilis and MPX in a non-virologically suppressed AIDS patient. The aim of this case report is to identify MPX as an emerging infectious disease and recognize it at an early phase, which would prompt the initiation of treatment early in the disease course to prevent severe complications in individuals that are notably at high risk.

This article was previously presented as a meeting abstract at the 2023 ACP DC Chapter on May 6, 2023.

## Case presentation

**Figure 1:**
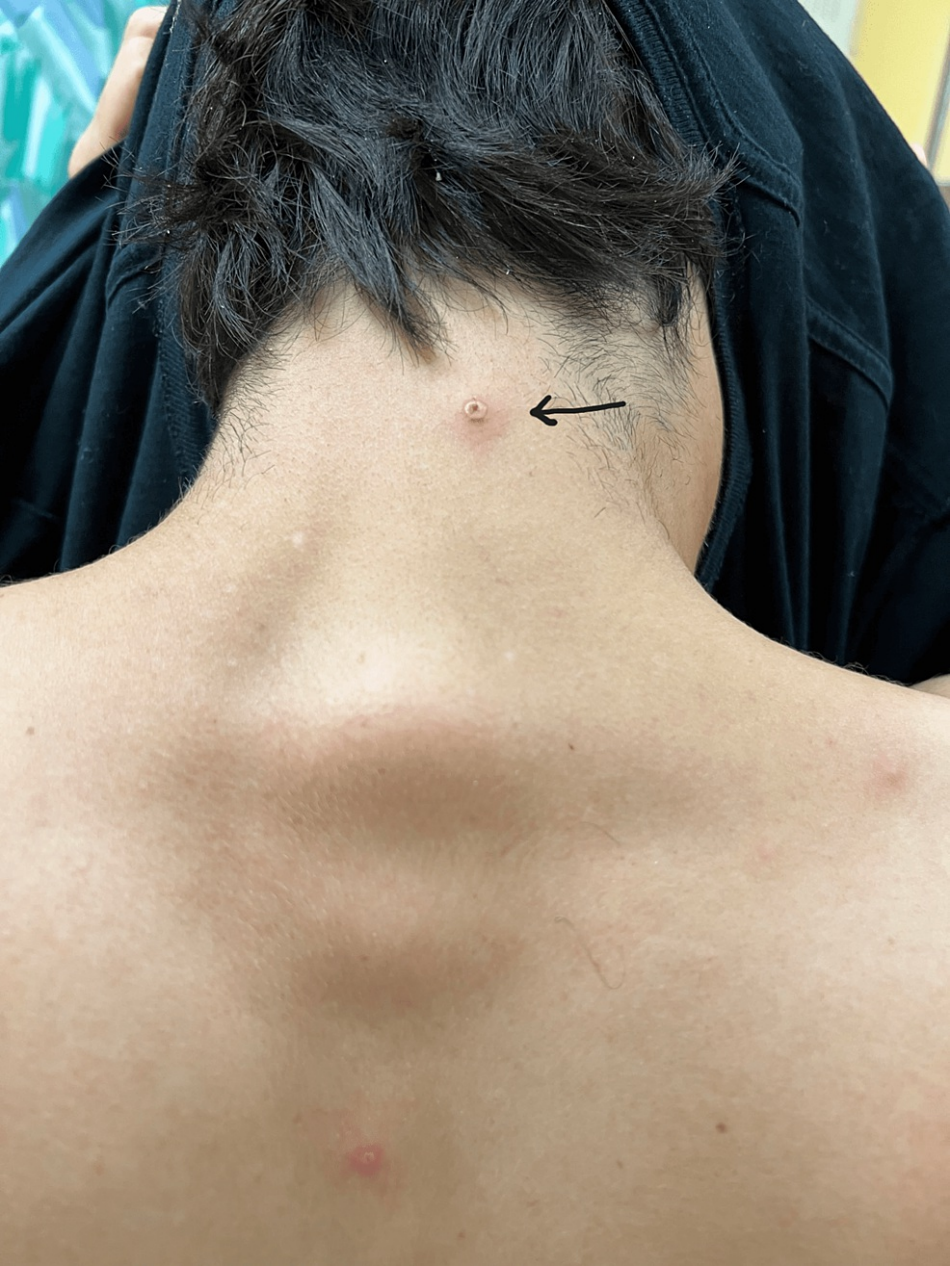
Key characteristics of monkeypox rash; neck and back

**Figure 2:**
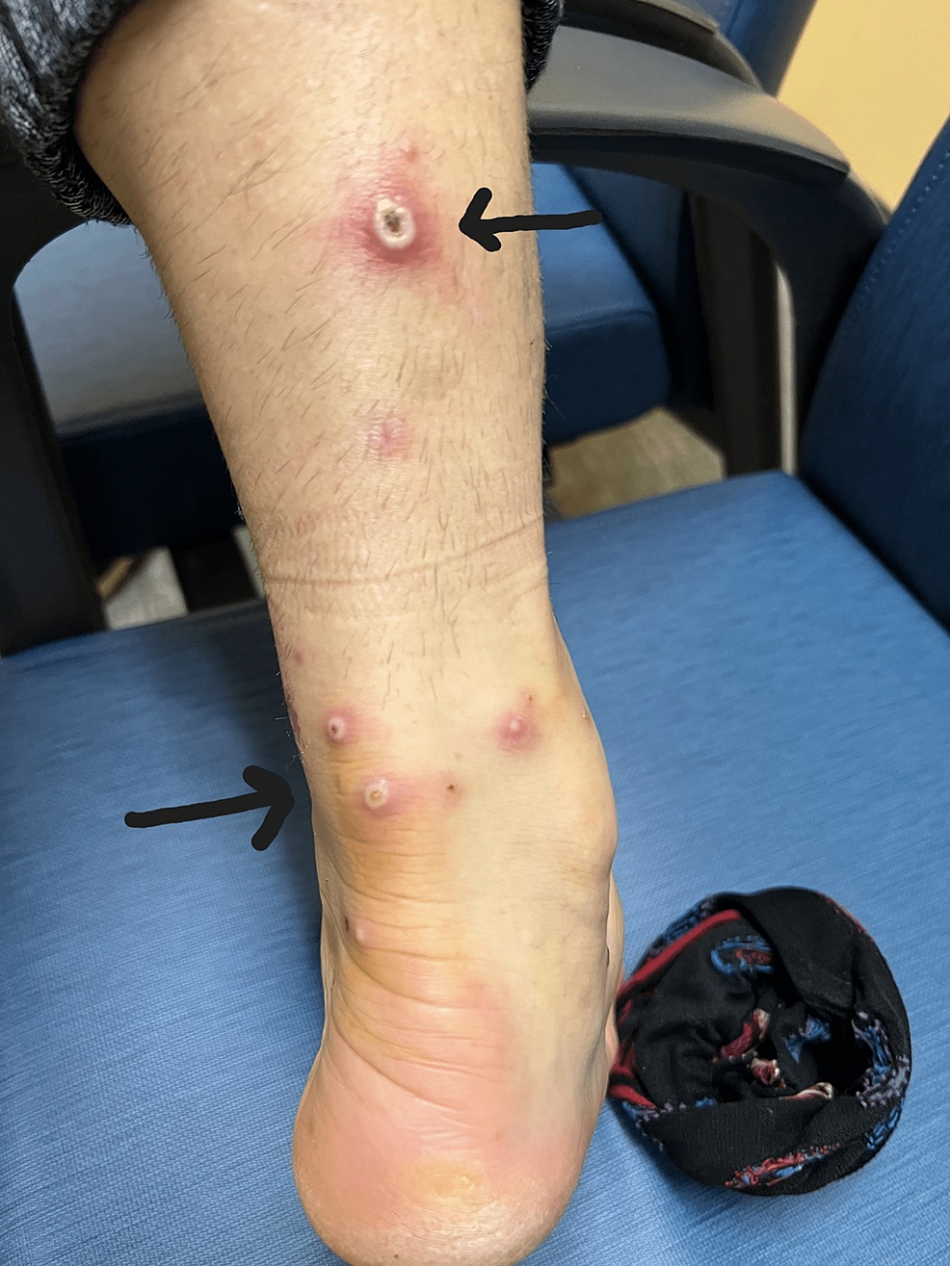
Monkeypox rash; leg

**Figure 3:**
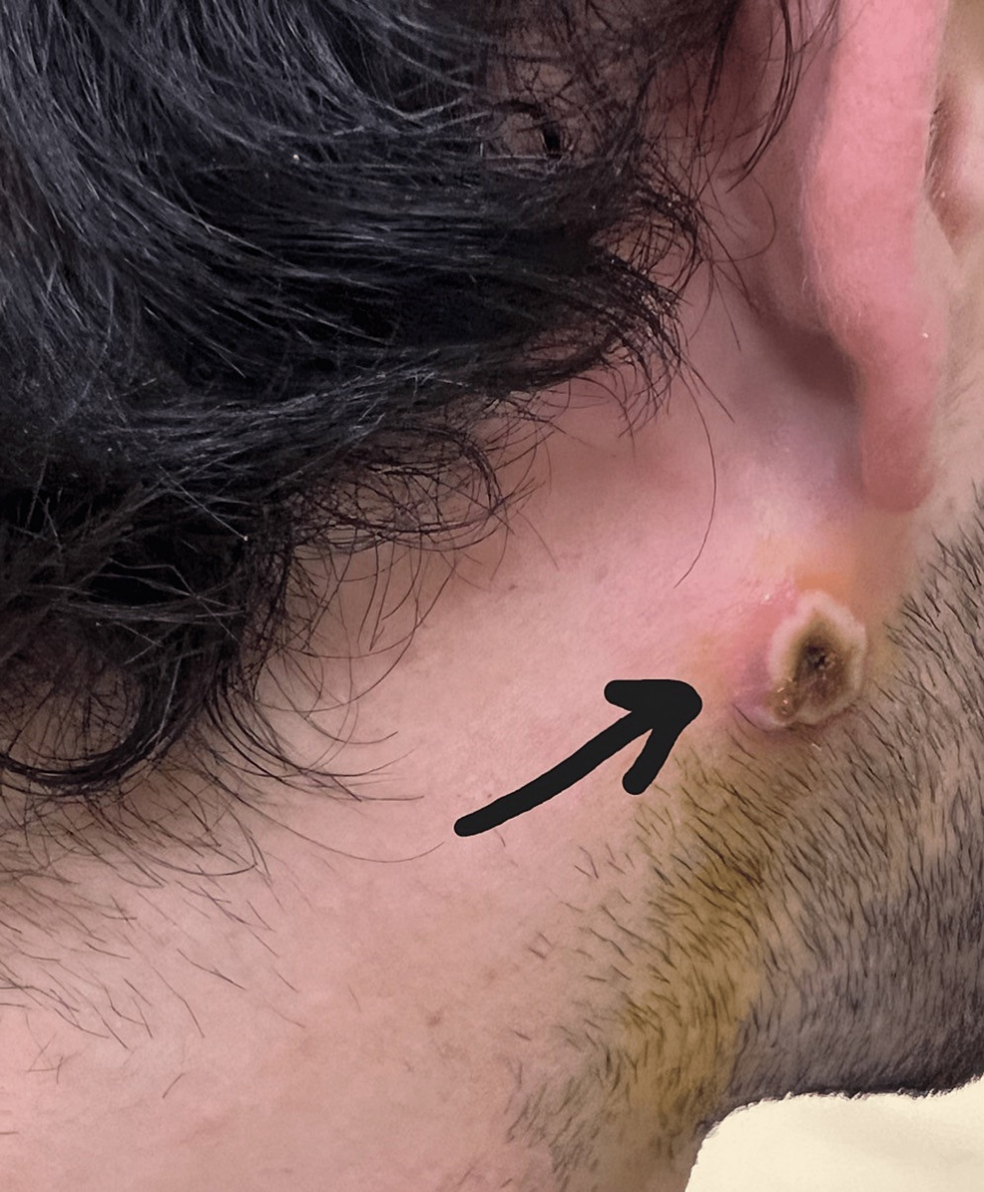
Monkeypox rash; neck

**Figure 4:**
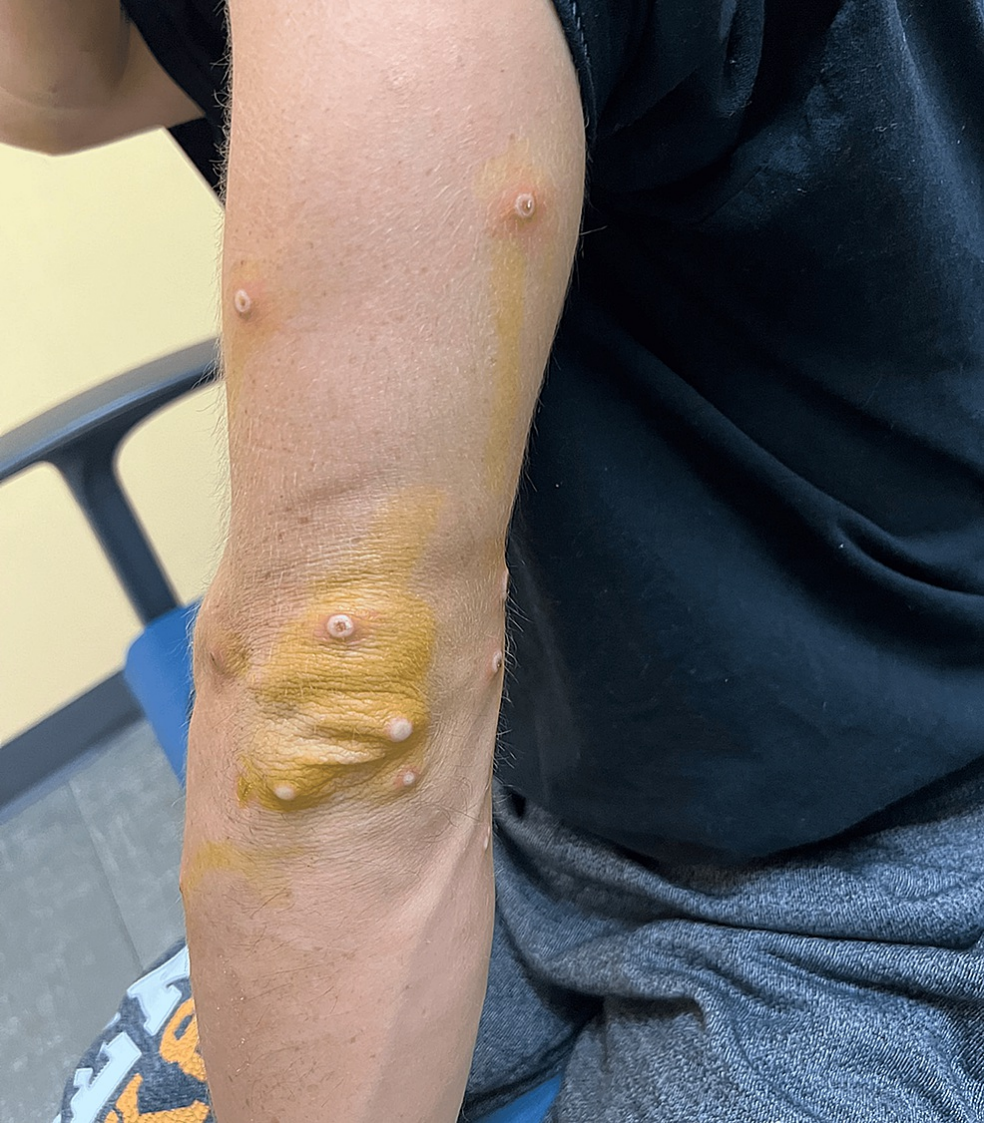
Monkeypox rash; arm

**Figure 5:**
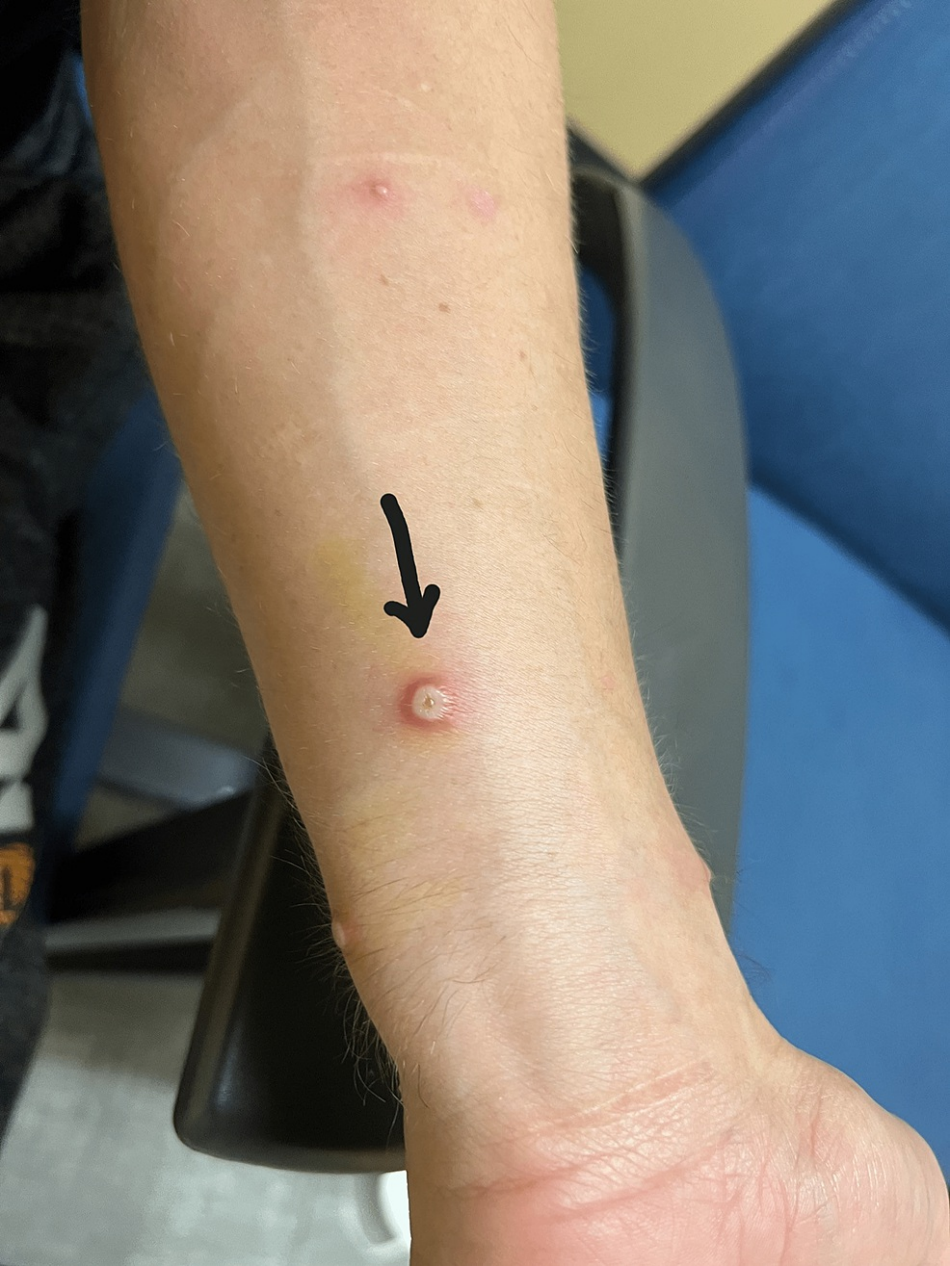
Monkeypox rash; arm

A 35-year-old male with HIV/AIDS, poorly adherent to anti-retroviral therapy, and penicillin anaphylaxis, recently treated for gonorrhea, presented with a widespread painful skin rash, swollen neck glands, fever, and generalized malaise. Two weeks ago, he noticed sparse lesions on his feet and assumed they were mosquito bites. Two days later, he saw they had some clear content; almost a week after the presentation, the rash had spread over the body. The exam was notable for numerous maculopapular and pustular lesions on the extremities, trunk, pustules on the penile shaft, and anal area with bilateral cervical and inguinal lymphadenopathy (Figures 1-5).

There were no significant neurologic symptoms or cranial nerve abnormalities. Labs were significant for an absolute CD4 cell count of 17 cells/mm3, a viral load of 1,290,000 copies/mL, a CD4/CD8 ratio of 0.01, a WBC count of 6,000/mL, an eosinophil count of 7.6%, an absolute eosinophil count of 0.65, normal liver tests, serology for herpes, cryptococcus negative, nucleic acid amplification test (NAAT) testing for sexually transmitted infections (STIs) such as chlamydia and gonorrhea was negative, and hepatitis C antibody positive with RNA in the normal range. Serology was positive for RPR 1:64 with reactive fluorescent treponemal antibody absorption (FTA-ABS).

The patient was started on treatment with doxycycline for secondary manifestations of syphilis. In the current situation, considering his risk factors, there was high clinical suspicion of other differentials for rash, such as MPX. Further inquiry revealed the patient’s roommate was diagnosed with MPX a week ago, with whom he had shared male partners. Furthermore, the patient has been deemed a suspected case with the characteristic new rash and has met the epidemiological criteria of having contact with a person with a similar-appearing rash. Hence, the Department of Health was contacted. Samples for orthopoxvirus DNA PCR were collected by dermatology. Tecovirimat (TPOXX) was approved under the non-research expanded access Investigational New Drug (EA-IND) protocol, also called "compassionate use," for primary or early empiric treatment. He remained in the hospital until the completion of treatment to monitor for signs of severe infection such as bleeding, sepsis, or encephalitis. Despite the complex nature of the co-infection, the patient responded well to therapy and showed signs of improvement.

## Discussion

MPX virus belongs to the orthopox genus of the poxviridae family. The first case of a human infected with MPX was discovered in 1970 and identified in the Democratic Republic of the Congo; new cases were later documented in Sudan and nearby countries [2]. The route of transmission is through close contact with skin lesions, respiratory secretions, sexual transmission, or recently contaminated objects [3]. People with MPX present with a rash that may be found on their hands, feet, face, chest, or genitals. The incubation period is 3-17 days. The rash presents in various stages, with a macular, papular, pustular, and vesicular appearance before scabbing over and desquamation. Constitutional symptoms include fever, headaches, lymphadenopathy, and a distinct rash [4].

Individuals with HIV can be at augmented risk of severe disease with MPX associated with an immunosuppressive state, including secondary bacterial infections and sepsis. Occasionally, co-infections may perhaps mask the symptoms of MPX. In addition, given the different stages of lesions, the condition can be confused with multiple other disease entities, including syphilis, scabies, herpes simplex virus, and molluscum contagiosum [5], with overlapping signs and symptoms [6]. Cases of multiple co-infections with MPX have been documented in the literature [7]. According to Angelo et al. [8], patients with HIV had a higher rash burden than patients without HIV, but no differences were identified in the proportion of men who had severe illness by HIV status. Of the 193 patients who were tested for STIs, 3% had primary or secondary syphilis, 15% had a concurrent infection, most frequently gonorrhea, and one patient had two STIs [8]. Therefore, to confirm a diagnosis in patients presenting with a rash, clinicians should obtain a comprehensive history for epidemiologic risk factors, assess for potential exposures, conduct a complete physical exam, perform appropriate lab tests, include a wide-ranging differential for other rash illnesses, and treat for co-infections concurrently.

Tecovirimat (TPOXX) is an antiviral drug used in treating MPX patients that was originally formulated for smallpox patients. It was indicated for this patient in the setting of an immunosuppressed state and disseminated disease [9].

## Conclusions

Prompt identification and timely treatment have led to appropriate care for this patient. Screening for co-infections is highly beneficial. The significance of this report is to highlight the importance of screening for STIs in key populations, along with an approach to other differentials in patients diagnosed simultaneously with MPX. Further studies are needed to determine the need for routine screening for MPX in high-risk populations. It is necessary to ensure accessible, rapid, and reliable tests to prevent the further spread of the disease.
